# Analysis of home-based rehabilitation in patients with motor impairment in primary care: a prospective observational study

**DOI:** 10.1186/s12877-017-0526-0

**Published:** 2017-07-14

**Authors:** Francisco Antonio Vega-Ramírez, Remedios López-Liria, Genoveva Granados-Gámez, Jose Manuel Aguilar-Parra, David Padilla-Góngora

**Affiliations:** 1 Complejo Hospitalario Torrecárdenas. Servicio Andaluz de Salud, Carretera del Sacramento s/n. La Cañada de San Urbano, 04250 Almería, Spain; 2 Department of Nursing, Physiotherapy and Medicine, CERNEP. University of Almería, Carretera del Sacramento s/n. La Cañada de San Urbano, 04250 Almería, Spain; 3 Department of Nursing, Physiotherapy and Medicine University of Almería, Carretera del Sacramento s/n. La Cañada de San Urbano, 04250 Almería, Spain; 4 Department of Psychology, University of Almería, Carretera del Sacramento s/n. La Cañada de San Urbano, 04250 Almería, Spain

**Keywords:** Primary health care, Geriatrics, Multimorbidity, Physiotherapy, Home care, Home based rehabilitation

## Abstract

**Background:**

The purpose of health and social policies is to encourage older people more longevity, remain free of disability and experience quality of life while living in their homes.

The aim of this study was to describe the characteristics of 473 patients diagnosed with motor impairment in primary care, the objectives of home-based rehabilitation and its functional impact.

**Methods:**

This prospective observational study was conducted in the Almería Health District. The analysed variables included age, gender, secondary diagnosis, Barthel Index (BI), physiotherapeutic objectives and techniques, and number of sessions.

**Results:**

The sample had a mean age of 83 years, and 59% were women. The assessed conditions with a high prevalence included osteoarticular pathology (55%), Alzheimer’s disease (15.1%), cardiovascular disease (13.7%) and stroke (6.5%). The techniques applied mainly consisted of functional exercises (57.1%), caregiver education (13.8%), and technical assistance (5.7%). There were statistically significant differences (*t* = −15.79; *p* < 0.001) between initial (*X* = 34.8) and final BI (*X* = 48.1), with an improvement of 13.4 points in patients’ functional capacity (95% confidence interval [*CI*]: −15.0 to −11.7). An equation was constructed to predict patients’ final BI as a function of the initial BI using a multiple linear regression model. The regression model explained 78% of the variance in patients with motor impairment.

**Conclusions:**

Important improvements were obtained in terms of functional capacity with a mean of ten sessions of physiotherapy. Lower patient age was correlated with higher initial and final functional capacities in primary care. This study aimed to present a useful starting point for decision making among management and health administration regarding this population group by approaching the process from the reality of practice and in relation to the rehabilitation provided.

**Trial registration:**

ClinicalTrials.gov identifier: NCT02715245; Date of registration: 18 January 2016.

## Background

Common challenges for the current health policies worldwide include the ageing population, the increasing prevalence of evolving chronic diseases and the care of people with multimorbidity and dependence [1, 2]. The maintenance of physical function in these people determines their clinical vulnerability and is critical for preventing hospitalizations, unnecessary admissions to nursing homes, consumption of social and health resources and mortality [2–5].

To plan these persons’ care, it is necessary to consider the prevalences and social and demographic aspects, along with the capacity of existing resources [6, 7]. At the same time, the health system should be organized based on the best available evidence regarding the needs of patients and their family [1]. In recent years, it has been emphasized that in all multidisciplinary social health interventions in older persons, the impact of functional status and the capacity to prevent dependence should be measured [8].

Immobility due to any medical diagnosis is one of the circumstances that can lead to increased loss of autonomy in older persons; several studies have emphasized a strong relationship between inactivity, loss of strength and muscular weakness [9–11]. When an older person is bedridden as a result of disease, appropriate physiotherapy techniques adapted to the patient’s stage are needed to achieve previous levels of function, prevent motor impairment, enhance activities of daily living (ADLs) and improve health status [4, 12].

The ideal place for such rehabilitation is the home, both for the older adult’s personal preferences and for the provision of care focused on emotional (a sense of familiarity that can be very comforting), social and community contexts (through the support of relatives and caregivers and access to nearby health and other community services) [13]. To meet these needs, the Andalusian Health Service and Mobile Rehabilitation and Physiotherapy Teams (MRPTs) were created as an intervention that acted exclusively in patients’ homes [14]. The effectiveness of these interventions in Spain for various processes (total knee replacement, stroke, chronic obstructive pulmonary disease, hip fracture or replacement) has been described in the scientific literature, showing improvements in patient functionality (independence in ADLs), quality of life, and satisfaction with the rehabilitation received [8, 12, 15]. HBR programmes are at least as good as inpatient postoperative rehabilitation programmes in terms of achieving functional outcomes for patients (including pain, functionality, walking, and balance) [15].

However, studies that provide evidence on home-based rehabilitation (HBR) in motor impairment or the sequelae after immobility in chronic patients with multimorbidity are insufficient, and thus more studies on this topic are needed to plan health services [16]. Knowledge of the patient group and the interventions targeting this group is essential, as this understanding can help achieve increased function of older people (preservation of mobility), reduce their physical, emotional and social problems (prevention of decline in daily activities, re-hospitalizations, and unnecessary referrals to nursing homes) and consequently, given its potential to generate savings, contribute favourably to the financial effects attributed to disability in this important sector of the population [1]. Currently, the complex chronicity and emergent social challenges lead to questions regarding the validity of health services (standard outpatient rehabilitation in a hospital setting or primary health care centres), while alternative care models such as HBR have been developed.

It is necessary to have a network of primary care and homecare programmes that can implement prevention and education initiatives to meet the health needs of this population. In addition, these efforts require the support of closely coordinated specialized services to reduce the impact of the consequences of immobility and caregiver overload or other situations, such as when patients arrive at home after hospital discharge. Thus, an action plan with early intervention should be proposed before situations of special clinical frailty in all fields and, in terms of coordination, between primary and specialized care in particular [4, 17]. Similarly, it should be considered that the ultimate purpose of interventions for chronic patients is to change the natural course of their pathology by delaying progression and improving the overall level of functionality and health based on a model of care that requires prior planning in the decision-making process [18]. However, the provision of these services is not the same throughout the Spanish territory due to the different health policies of each community and the issue of geographical dispersion [15].

HBR teams often report barriers and difficulties applying an appropriate care model, which can require new areas of clinical management training. Determining the most appropriate objectives and techniques for patients’ treatment, as well as understanding the impact of HBR on the results, are two key priorities considering the importance of optimizing the treatment and diversity of resources available for this purpose.

This article analyses the home-based rehabilitation that MRPTs provide as a response to the needs of a sample of older people with motor impairments, describing their characteristics and the functional effects after treatment is applied. We hypothesized that a referral to home rehabilitation improves their level of disability in terms of improvement in functional recovery, as assessed by the Barthel Index.

## Methods

**Fig. 1:**
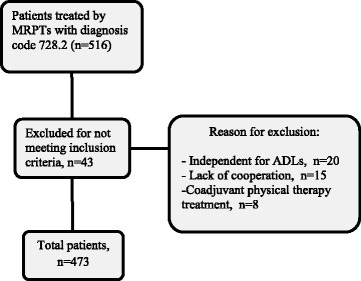
Flow of the patients through the study

This prospective observational study was conducted with 473 patients treated by MRPTs of the Almería Health District in the period from 2009 to 2014 (Fig. 1).

*Inclusion criteria*: patients with a principal diagnosis of code 728.2, determined by a rehabilitation physician according to ICD-9 coding (muscular wasting and disuse atrophy), including patients with substantial functional deficits, motor impairment, muscular atrophy, or post-immobilization sequelae, who were referred to MRPTs due to the presence of structural barriers (to facilitate the treatment of patients who could not visit the hospital due to stairs or other obstacles in their homes) and patients with multimorbidity (having a medical diagnosis that determined functional impairment; reintegration in the home; medical instability among older people or a state of being home bound) were eligible [14].

*Exclusion criteria*: patients who were independent in ADLs, were not accepting treatment, did not cooperate or had caregivers who did not cooperate, and received adjuvant physiotherapy at a different institution were excluded.

The analysed variables in each patient were age, gender, secondary diagnosis, functional capacity in ADLs assessed using the Barthel Index (BI) [19], physiotherapeutic objectives, rehabilitation techniques applied and the number of treatment sessions.

The intervention: the personnel and organization of MRPTs [14] are summarized in Table 1. Healthcare providers typically identified the factors related to risk of falls, treated the existing limitations in joint movement (by means of passive movilization and stretching), and mitigated muscular atrophy (with active-assisted or active and strengthening exercises, for example, isometric or isotonic exercises), by prioritizing the training and improvement of gait and balance.

**Table 1:** Brief description of the personnel and organization of MRPTs [14]

Mobile rehabilitation and physiotherapy teams were born in 2002 within the Aid Plan for Andalusian Families. Decree 137/2002, April 30.	- Andalusía would have 46 home rehabilitation and physiotherapy teams in all of the provincial capitals and the Campo de Gibraltar. - In Almería: four basic teams have remained until today; 41,582 inhabitants per team.
The target population and coverage area of MRPTs	- Physically disabled patients - Primary caregivers of patients in a family environment - Professionals of basic primary care teams
Composition of support devices for rehabilitation in Primary Care from 2002 to the present, in the Almería Health District	- One rehabilitation physician - Four physiotherapists - One occupational therapist - Two guard drivers The intervention of the team comformed by physician, physiotherapists and occupational therapist is scheduled on morning or afternoon shift, accessing the patient’s home in one of the two cars that are driven by guard drivers.
Available equipment	- Two cars - Portable equipment: Transcutaneous neuromuscular stimulator and ultrasound Most techniques are based on kinesitherapy or manual therapy in the patient’s home.
Number of patients per professional/day	Between 5/7 patients daily, depending on the geographic dispersion of homes.
Number of sessions per patient	Stipulated sessions should not exceed 3 weeks or 15 sessions. In exceptional cases, continuation will be assessed by the rehabilitation physician.
Completion of service	When the patient can move to a physiotherapy room; worsening of health status that contraindicates therapy; functional recovery of the patient; when the caregiver has been instructed in patient management; when the number of stipulated sessions has been exceeded.
List of processes for home based rehabilitation treatment	Musculoskeletal disorders; traumatic orthopaedic pathology; lower limb amputation; neurological, respiratory and cardiovascular diseases.

Family and caregivers received training to promote independence of the patient and were also given information about the intervention regarding the patient and its objectives, helping them incorporate these activities into their daily routine. Additionally, they received information on health and community resources to help them take care of their own physical and mental health. Furthermore, they were encouraged to participate in support groups for stress management, as well as other activities.

All patients provided written informed consent before treatment in accordance with the Helsinki Declaration.This study was approved by the scientific ethics committee of the Torrecárdenas Hospital Complex (Almería) and the research commission of the Almería Health District (CEIC-AL 39/2012) and adhered to guidelines of the International Committee of Medical Journal Editors. Our study was based on data from a randomized controlled trial of a Home-based Rehabilitation Programme in Multiple Chronic Diseases (RCT registered at ClinicalTrial.gov; NCT02715245) and a research project: PI 0354/2014.

### Statistical analysis

Descriptive and bivariate analyses, seeking possible associations between dependent and independent variables, were performed using the statistical program SPSS version 22. Additionally, analysis of variance (*ANOVA*) was applied for the initial and final BI values and for patients’ age by age groups; to determine the validity of this technique, the randomness of the sample was checked (Rachas test; independence of variables; normality; and homogeneity of the variances with the Levene test). If all criteria were met, multiple comparisons were made using the Bonferroni test; when homogeneity was not fulfilled, Dunnett’s T3 test was performed. Relationships between quantitative variables were assessed through correlation measurements (Pearson linear), and the regression line that was obtained determined the predictive model in relation to the initial and final BI values of the patients.

## Results

The mean age of the 473 selected patients was 83 years (standard deviation [*SD*] = 8.1), and 59% were women. The reasons for referral to the unit were being a patient with multimorbidity or having advanced age with a risk of comorbidity (75.3%) and having structural barriers in the home (24.7%).

The most common conditions that were referred to the service (secondary diagnosis) were osteoarticular pathology (55%), Alzheimer’s disease (15.1%), cardiovascular disease (13.7%), stroke (6.5%), chronic obstructive pulmonary disease (3.6%), Parkinson’s disease (3.6%), and amputation (2.5%).

The main treatment objectives were to achieve the highest possible functionality (41.9%), offer education to the caregiver (28.5%), administer gait training (20.9%), provide pain relief (7.2%) and increase breathing capacity (1.5%). The different rehabilitation techniques applied were grouped into five categories: functional exercises (57.1%), functional exercises and electrotherapy (6.6%), caregiver education (13.8%), functional exercises and caregiver education (16.9%), and adaptation or technical aids (5.7%). The mean number of physiotherapy sessions was 10.1 (*SD* = 9.2).

The difference between the means of the initial (*X* = 34.8; *SD* = 26.2) and final (*X* = 48.1; *SD* = 33.0) BI values of this sample was statistically significant according to Student’s *t* test for related samples (*t* = −15.79; 95% confidence interval [*CI*]: −15.0 to −11.7; *p* < 0.001). In Table 2, the initial and final BI values by secondary disabling process are also described.

**Table 2:** Initial and final Barthel Index by secondary disabling process

Secondary diagnosis	Initial BI	Final BI	Statistic	p	d
	$\overline{X}$ (SD)	$\overline{X}$ (SD)	T		
Osteoarticular pathology	36.6 (26.0)	50.3 (33.3)	−11.78	<0.001	0.459
Alzheimer Disease	18.2 (18.5)	26.3 (27.0)	−4.02	<0.001	0.351
Cardiovascular Disease	44.2 (28.4)	58.20 (28.8)	−6.09	<0.001	0.486
Stroke	35.5 (23.3)	58.9 (32.6)	−6.69	<0.001	0.827
Chronic obstructive pulmonary disease	47.1 (26.8)	59.6 (27.8)	−4.33	0.001	0.456
Parkinson Disease	42.8 (24.7)	57.1 (30.2)	−4.17	0.001	0.520
Amputation	15.5 (14.0)	22.2 (21.8)	−1.78	0.111	0.363

Statistically significant differences were found in the number of physiotherapy sessions, showing that women received fewer sessions than men (*t* = −2.09; 95% *CI*: -3.5 to −0.1; *p* = 0.037). An analysis was performed on quantitative variables (age, initial BI, final BI, number of sessions), with the results differentiated by gender to test whether there were differences between genders (Table 3).

**Table 3:** Student’s T test for independent samples (gender)

Variables	Female	Male	Statistic	*p*	95% *CI*	*d*
	$\overline{X}$ (SD)	$\overline{X}$ (SD)	T			
Age	83.6 (8.2)	82.4 (8.1)	1.58	0.115	−0.29 / 2.70	0.137
Initial barthel index	33.7 (25.4)	35.9 (27.1)	−0.90	0.367	−7.01 / 2.59	−0.084
Final barthel index	46.7 (32.6)	50.2 (33.6)	−1.13	0.256	−9.72 / 2.59	−0.105
Physiotherapy sessions	9.4 (8.2)	11.2 (10.5)	−2.09	0.037*	−3.50 / -0.10	−0.157

Note: *P < 0.001

X = mean

The differences between groups of patients in different age groups (60-69; 70-79; 80-89; 90-99 years) were also analysed in terms of the mean initial and final BI values, and there were statistically significant differences between the two Barthel index assessments in every age group (*p* < 0.001). Analyses were also performed to check whether the groups fulfilled the applicability hypothesis; in the case of initial BI, all conditions were met for applying ANOVA, which indicated that there was a statistically significant difference between groups (*F* = 4.89; *p* = 0.002). After analysing data with the Bonferroni post hoc test (difference of means I-J = 13.2; *p* = 0.002), a difference was found between the age group of patients 70-79 years old (*X* = 41.1) and the age group of those 90-99 years old (*X* = 27.9).

The final BI data did not fulfill the hypothesis of homogeneity of variances, and the Brown-Forsythe test (*p* < 0.001) was thus performed. Based on Dunnett’s T3 post hoc test, there were statistically significant differences between the age group of patients 90-99 years old and the age groups of patients who were 60-69 years (difference of means I-J = 27.4; *p* = 0.002), 70-79 years (difference of means I-J = 21.8; *p* < 0.001) and 80-89 years (difference of means I-J = 13.3; *p* = 0.003).

Final BI was directly correlated with initial BI (strong correlation) and was inversely associated with age but did not show correlations with the number of sessions (*r* = 0.085; *p* = 0.115). Pearson correlations for the quantitative variables age, initial and final BI, and number of sessions are presented in Table 4.

**Table 4:** Correlations between age, initial Barthel index, final Barthel index and the number of treatment sessions

		1	2	3
1. Age		-		
2. Initial Barthel Index	*Pearson’s Correlation*	−0.170^**^		
	*Significant*	0.001		
	*N*	357		
3. Final Barthel Index	*Pearson’s Correlation*	−0.289^**^	0.754^**^	
	*Significant*	< 0.001	< 0.001	
	*N*	345	345	
4. Number of treatment sessions	*Pearson’s Correlation*	−0.243^**^	−0.179^**^	0.085
	*Significant*	< 0.001	< 0.001	0.115
	*N*	357	356	344

Note: **. The Correlation was statistically significant *P* < 0.001

Table 5 shows that final BI was inversely related to age (β = − 24, *p* = 0.010), i.e., the lower the age of the patient, the higher the final BI score. Additionally, the higher the number of rehabilitation sessions (β = 0.88 *p* < 0.001) and the higher the initial BI score (β = 1.02 *p* < 0.001) was, the higher patients’ final BI score was. The regression model explained 78% of the variance in final BI in patients with motor impairment.

**Table 5:** Multiple linear regression model. Final BI as a function of age, initial BI and number of sessions in patients with motor impairment

	Final barthel index		
Variables	Beta	t	*p*
Age	−0.24	−2.81	0.010
Physiotherapy sessions	0.88	10.68	< 0.001
Initial barthel index	1.02	36.01	< 0.001
(R) = 0.88;	(R2) = 0.78		

Finally, the following equation was constructed to predict final BI as a function of age, initial BI and number of sessions using a multiple linear regression model (Table 5):

$$ \mathrm{Final}\ \mathrm{Barthel}\ \mathrm{Index}=23.41\hbox{-} 0.24\ \mathrm{x}\ \mathrm{age}+0.88\ \mathrm{x}\ \mathrm{number}\ \mathrm{of}\ \mathrm{sessions}+1.02\ \mathrm{x}\ \mathrm{Initial}\ \mathrm{Barthel}\ \mathrm{Index} $$

## Discussion

This study on MRPTs analysed their clinical activity and the treatments provided to mostly chronic patients with motor impairments. The findings corroborate the results from research that explains how multidisciplinary HBR with a focus on function (small gains in ADLs or an “aging at home” strategy) is able to improve initial level of functioning and independence among older persons in frail health [4, 20–22].

The process of allocating patients in HBR is influenced by their needs, such as ADL restrictions and home safety concerns [23]; this care should be based on the patient’s own perspective and goals [24]. Appropiate models of funding and service delivery should be developed to ensure that patients have access to rehabilitation that aims to prevent deterioration by integrating these practices for chronic, medically complex long-stay clients in the community [23]. This information could be useful as a basis for designing local strategic plans that facilitate the provision of higher quality primary care home services.

More than half of the patients treated by MRPTs were women, with a mean age of 83 years; comparisons of these results with other studies [25, 26] showed that the age of patients with multimorbidity is usually between 66 and 82 years.

In this study, three-quarters of the patients had life-threatening comorbidities (patients who had substantial functional deficits or were home-bound), with osteoarticular and neurological diseases constituting high percentages. It worth noting that this type of patient does not appear suddenly; rather, these patients tend to present first as potential candidates for this type of rehabilitation, exhibiting on-going symptoms and obvious impairment. The low initial Barthel score was expected, as patients tended to be older and to have more co-morbid conditions, which may influence their functional outcome at discharge. Earlier intervention at home could provide significantly better results in terms of physical function, disability and even quality of life.

The main applied treatment in the patient’s home was functional exercises, which aimed to achieve the highest possible functionality, as well as gait training. On rare occasions, electrotherapy was used, normally for analgesic purposes. In more than 25% of cases, health education was provided to families and caregivers. Thus, in accordance with patients’ condition, their tolerance of activities and their ability to independently exercise self-care, interventions in HBR are implemented to improve sequelae derived from motor impairment or functional limitations.

Considering its importance, the high improvement in functional capacity obtained with a mean of ten sessions of physiotherapy treatment in this study is worth noting. In other regions (Catalonia, Spain), HBR patients received a similar average of nine sessions per year, although with great variability. The number of sessions was greater for patients who were dependent in ADLs, and who had bed sores or social problems [3]. The group of older patients was found to start with a lower BI than patients who were in the 70 to 80 year range, and this difference was maintained in the final BI. A lower patient age was correlated with higher initial and final functional capacities. A study by García-Morillo [26] highlighted that 16% of patients with multimorbidity in their sample showed a difference of 10 points between their baseline and discharge BIs. The predictive model generated in this investigation allowed us to estimate the final BI that a patient could achieve based on their age, initial BI and number of treatment sessions. Previous studies on HBR [12] have shown that the disabling condition for which the patient is referred could be related to the number of treatment sessions required. This information can be considered to be consistent with the results of this study, which show that women required a lower mean number of sessions of physiotherapy treatment; this finding could be due to the unequal distribution of men and women in the disabling processes secondary to the post-inmobilization sequelae.

This study found that the lowest initial Barthel Index was obtained for patients with Alzheimer’s disease, amputation and stroke effects, whereas patients with chronic obstructive pulmonary disease and cardiovascular disease reached the highest scores. Significant improvements were confirmed in pre- and post- Barthel Index scores for each of the abovementioned diagnoses, showing greater functional independence of patients with stroke, Parkinson’s disease and cardiovascular diseases. The maintenance of these patients’ mobility is essential to preventing a decrease in their ADLs as well as re-hospitalizations. Therefore, helping families by providing support and thus keeping the patients at home longer is essential (preventing unnecessary remissions to nursing homes).

The main limitation of this study is the limited ability to generalize of the results to other regions, because the selection process did not generate a representative sample of patients receiving HBR in Andalusia. Furthermore, the study was performed in a context where the services provided varied greatly from some professionals and others. However, the selection and involvement of a large number of patients over a long period of time and the quality of the data collected provided a good description of the current situation in the province. The importance of HBR and the preference of the population to receive this intervention in the home justified an analysis of the situation regarding the dependent population in primary care; the findings could help create new programmes or to adapt existing ones to the patients’ sociodemographic and clinical characteristics. Because of the need for progress on proposals to improve HBR, it is necessary to start from epidemiological and descriptive studies that facilitate the identification of groups of patients with different levels of need and that help guide and adjust assistance to the intensity and type of treatment that each group requires [27].

## Conclusion

In conclusion, this study provides a first approach to investigating patients with motor impairments from the point of view of existing MRPTs in primary care. Additionally, this study aimed to present a useful starting point for the decision making of management and health administration regarding this population group by approaching the process from the reality of practice and in relation to the rehabilitation provided. Important improvements were obtained in terms of functional capacity with a mean of ten sessions of physiotherapy in patients with motor impairments. Lower patient age was correlated with higher initial and final functional capacity scores among patients in primary care. Professional teams must adapt to the needs of the people, which requires objective and critical analyses of the effectiveness of activities, as well as openly reconsider their services with new organizational approaches to optimize the resources and resolution capacity in primary care.

## Abbreviations

ADLs: Activities of daily living
BI: Barthel Index
CI: confidence interval
HBR: Home based Rehabilitation
MRPTs: Mobile Rehabilitation and Physiotherapy Teams
SD: Standard deviation
